# Ageing-friendly cities for assessing older adults’ decline: IoT-based system for continuous monitoring of frailty risks using smart city infrastructure

**DOI:** 10.1007/s40520-019-01238-y

**Published:** 2019-06-21

**Authors:** Patricia Abril-Jiménez, Javier Rojo Lacal, Silvia de los Ríos Pérez, Miguel Páramo, Juan Bautista Montalvá Colomer, María Teresa Arredondo Waldmeyer

**Affiliations:** Escuela Técnica Superior de Ingenieros de Telecomunicación, Universidad Politécnica de Madrid, 108, Avenida Complutense 30, 28040 Madrid, Spain

**Keywords:** Aging, Early detection, Activity pattern, Data analysis

## Abstract

**Background and aims:**

Population ageing is a typical phenomenon of developed countries with a great influence in their economy and society, with an increment on age-related expenditures. Disruptive solutions are needed to deploy new cost-effective and sustainable solutions for aging well and independent living of our seniors. In this sense, new technological paradigms as IoT technologies and smart cities have the potential to become main drivers for innovation uptake. The purpose of this study is to describe a longitudinal cohort study in smart cities for assessing early frailty symptoms deploying an unobtrusive IoT-based system in the Madrid city.

**Methods:**

A system was deployed in the Madrid city with the participation of 45 elderly users for an average of 71 weeks. Metrics were assessed by the available sensors in combination with the open data infrastructure of Madrid. Metrics include activity of the user, weekly visits pattern and transport daily usage pattern. System engagement was also monitored. Participants are assessed bimonthly with health and functional questionnaires.

**Results:**

45 older adults with a mean age of 79.1 years. Participants activity patterns monitor detected changes during potentially risky situations that usually were not reported by traditional assessment tools. Analysis of data collected enabled to identify absence of frailty (robust or post-robust status)

**Discussion and conclusions:**

The results demonstrate the feasibility of engaging older adults with an IoT-based system and the successful collection of their activity metrics. Variation in the activity patterns may be a first sign of functional decline and enables to identify potential areas of early intervention.

## Introduction

Population of older adults in Europe is increasing. The impact over the public welfare and care systems is evident. Innovation in methods of managing ageing functional decline is critical in balancing the needs of older adults with limited health-care resources. In this sense, the concentration of population around cities offers opportunities that better manage dedicated aging resources [1].

In this sense, smart cities are a paradigm that can disrupt in the manner that European Union manages the public services to respond to the needs of its changing population, increasing the efficiency of the resources, while improve quality of life of citizens [2].

This document analyses the impact of a smart city on the early detection of clinical ageing-related events that could go unnoticed by the traditional health assessment tools in a very early stage. This is especially important for ageing syndromes, such as frailty, in which prompt detection can prevent, even reverse [3], this condition, improving life expectancies of the affected individuals. It is precisely the reversibility condition that makes frailty symptoms detection interesting, but the lack of a universal definition of frailty hampers practical implementations [4]. In general terms, frailty is a multi-dimensional clinical condition characterized by decreased physiological resilience and weakened response to stressors, so that the frail individuals are predisposed to adverse events such as hospitalization, institutionalization and mortality.

Physical, social and physiological factors’ influence on the frailty assessment scale, but, active individual has been related with lower rates of developing frailty compared with sedentary individuals [5]. It is noted physical activity is defined as any kind of muscle contractions and includes daily-life activities such as house work, walking, etc. The benefits of physical activity are not only evident in physical phenotype of frailty, they are also evident on cognitive and social frailty dimensions. Physical activity and exercise in older adults are associated with lower rates of cognitive impairment and depression (most common forms susceptible to aging) [6].

With all these evidences suggesting physical activity as frailty assessment element, we propose the validation of unobtrusive monitoring systems that take advantage from the citizen’s smartphone sensors (GPS, IMU, system clock, etc.), and the smart city infrastructure itself, such as public buses of Madrid open network, municipality’s open data, aims to conclude the individual frailty status using data gathered from older citizens daily activity while moving around the city, including individual’s activity patterns, visited places while they move in terms of public places like shops, health infrastructures, sport areas, etc. and private place, like relatives and friends’ home mainly and the use of the (public) transport.

## Research design and methods

The proposed system was deployed in Madrid City (Spain) and tested by Universidad Politécnica de Madrid (UPM), in collaboration with the Empresa Municipal de Transportes (EMT), partially owned by the Madrid Municipality, beginning in 2016. This initial system development was conducted in the Smart House Living Lab together the Campus Internacional de Excelencia de Moncloa [7], with the collaboration of ten senior volunteers of Unión Democratica de Pensionistas (UDP) and Seniors Españoles para la Cooperación Técnica (SECOT) which are retired older adults associations which usually participates in a variety of programs and research initiatives related to active and healthy ageing promotion and entrepreneurship. Within this group of seniors, we first examined the idea of the system, the feasibility, reliability, and usability of the technologies and proposed use cases, before the wider dissemination to the real deployment in the Madrid city.

### Participants

All the participants were recruited from the Madrid metropolitan area, using the Day Center Network of the Madrid Municipality and different seniors associations, UDP and SECOT, mainly. To facilitate recruitment, senior center administrators and association directors were contacted concerning the study and formal community presentation was conducted to enhance awareness of the project. Potential participants were recruited at the end of presentation and contacted by phone to be assessed at baseline. All of them were provided written informed consent before participating in the study activities. The protocol was approved by the UPM Ethical Committee. Enrollment began in December 2016 and continued on a rolling basis until April 2018.

The study population consisted of older adult citizens of Madrid City over 75 years old; moving independently around city, managing well (self-assessment) and without evident symptoms of frailty (1–3 in the frailty scale) [8] according criteria of the scale of the Law of Personal Autonomy and Care for Dependency of April 21 of the Spanish Government (Spanish Dependency and Autonomy Law) [9] relating to the treatment and protection of individuals in a situation of dependency.

### Protocol assessment procedure

Participants were assessed in-home at baseline, at 2 weeks intervals (by telephone) and during 2 months intervals in-home visits with research personnel who administrated standardized health and function questionnaires and physical and cognitive examinations.

Biweekly calls were oriented to perform a follow-up of the major health events (illness, hospitalizations, falls, sadness, poor appetite, etc.), social events (lack of leisure plans, changes in their leisure plans, friends relationships, etc.) and family events (deaths, moves, financial problems, etc.) that could impact in their frailty or MCI index. Calls were also oriented to collect experiences and satisfaction with the proposed technological solution and maintain engagement and motivation within the research.

The bimonthly assessment included an examination of the Spanish Dependency Assessment Law criteria using the medical histories, medication list and the completion of the Mini Cog [10], grip strength test [11] and Functional Ability Index [12] test. In addition to these assessments, participants were queried with regard to their experiences, aptitudes and beliefs about system monitoring using a 10-items questionnaire, based on [13, 14] that assessed their views of the IoT technology use and semi-structured interview oriented to discover the usefulness perception of the solution and issues of privacy and security. Health, cognitive, behavioral and functional assessments are summarized in Table 1.

**Table 1:** Assessment follow up schedule

Assessment	Bi-weekly	Bi-monthly
IoT usability Questionnaire	X	
Spanish dependency Assessment law criteria		X
Semi-oriented interview of solution usefulness and privacy and security	X	
Mini cog		X
Grip strength test		X
Functional ability index		X

The standardized conventional assessments were established to identify incidents of functional decline, risk of frailty and to compare the bimonthly-acquired measures with the automated continuous data gathering from the proposed system.

### Continuous pattern monitoring system

The proposed system collected data of the participants’ activity when moving around city. In particular the following data groups were collected: activity of the user (number of steps, distance covered, and walking average speed), weekly visits pattern (type of POI, number of visit per POI, and visit duration) and daily transport usage pattern (number of trips, used bus lines, distance, and time per trip). The system uses smartphone-embedded sensors (GPS, IMU, Bluetooth, and Wi-Fi), the communication network of the EMT buses, with information of the bus line, stops ID, time per trip, etc., the Madrid Municipally open data service with real-time information about traffic and urban links information, pollution, planned events, etc. and weather information of the Agencia Estatal de Meteorología (AEMET).

**Fig. 1:**
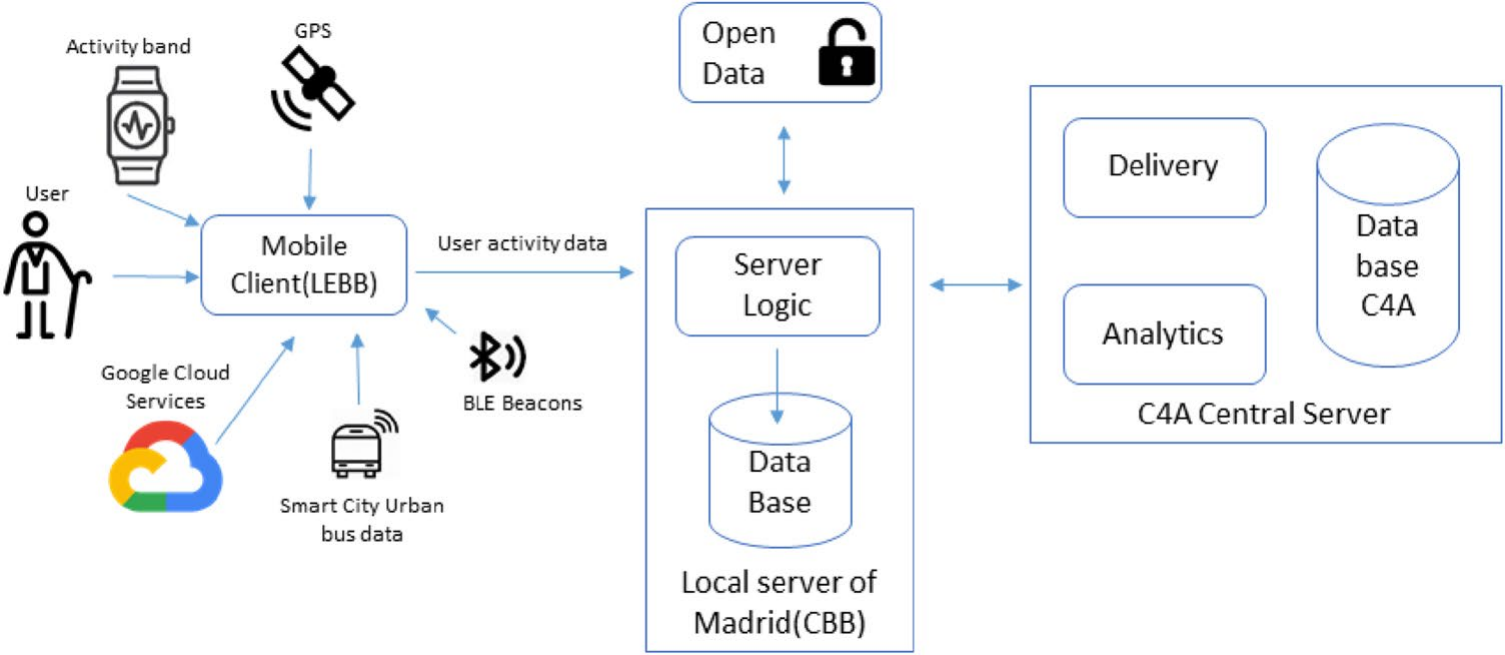
Architecture of the system deployed in Madrid

The proposed system consists of two main components, namely the Mobile Client (MC) and the Madrid Local Server (MLS) integrated with the C4A Central Server. The architecture of the City4Age global system and the concepts of LEAs and measures have been widely discussed in [15–17]. Figure 1 shows general Madrid pilot deployment components for the unobtrusive data collection from a heterogeneous sensing infrastructure.

**Fig. 2:**
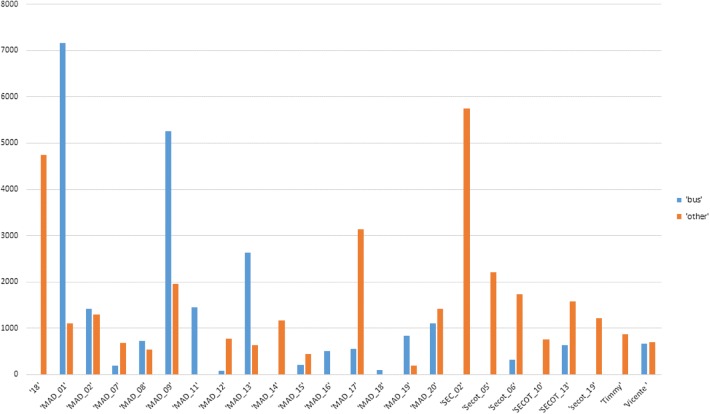
The bar chart showing the transport behavior data during one day in some of the participants. The data shows time expended in sec in each of the transport

**Fig. 3:**
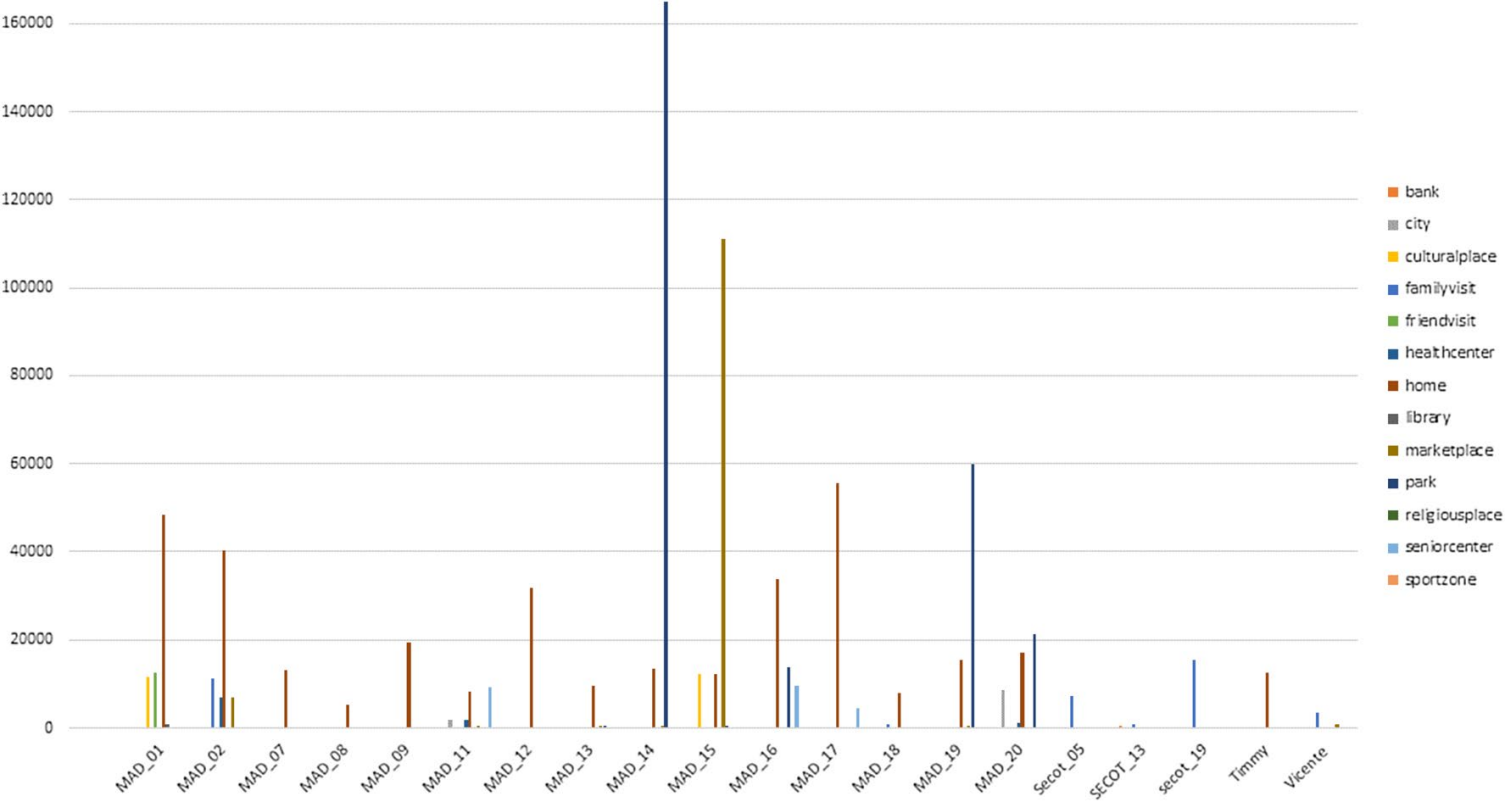
The bar chart showing the POIs expended time per type of POI and user during one day in some of the participants

The raw data coming from described sensors can be transformed into meaningful geriatric data that allow behavioral interpretation, namely Low Elementary Actions (LEAs). LEAs are the individual actions gathered from the user activity and are recorded in almost real time. A session of physical activity monitored includes at least two LEAs (starting and ending an activity), typically, BODY_STATE_START and BODY_STATE_STOP and, depending on the nature of the LEA, includes other records for proficiency describing the activity, such as a timestamp when the user enters and leaves a particular body state (i.e., still, walking, sleeping, etc.) and the POI_ENTER/POI_EXIT LEAs, indicating the location type and/or the GPS coordinates (by exploiting the GPS receiver of the smartphone). Resulted data help to identify elderly people behaviors and, above all, their variations, which may represent an early indicator of more severe conditions (Figs. 2, 3).

LEAs are sent to the Madrid Local Server (CBB). It is in charge to complete the data message object if any other information is missing, since the CBB has access to every data and logs coming from the smartphone of every univocal identified users. It is also responsible for collecting contextual information from open services such as regional weather conditions or public transport incidents by means of periodical queries to the Madrid Open Data Repository. CBB performs computations to calculate MEASURES based on the given LEAs.

Finally, all data collected (both MEASURES and LEAs) are uploaded periodically (typically daily) to the City4Age central repository for accomplishment of the full data processing and interpretation lifecycle. In this way, by automatizing the process of data gathering through unobtrusive technologies, it is possible to obtain data directly related to proper geriatric factors, allowing a fast, scalable and automatic functional decline risk detection for elderly people.

### Monitoring

Recruited participants have been provided with already pre-configured hardware needed to carry out the experiment: a pair of BLE beacons and a smartphone with the Madrid City4Age pilot application running. The two BLE beacons are required to discriminate home regions and one was placed at the building mailbox and the other in the entrance of the home. To asses behavioural patterns of the participants and to conclude social, physical and cognitive patterns, participants were asked to indicate all the activities they were doing and where they were usually doing. With these information, the smartphone applications were configured with each participant’s Point of Interest regions (POIs) that represent Madrid areas or places in which each particular participant usually does daily activities and we combined into nine categories: Park, Cultural Place, City Place, Familiar’s House, Friend’s House, Health Site, Marketplace, Senior Centre and religious place. Participants only should be aware enough to carry their mobile when going out of their homes and charging the battery every day.

During a maximum of 95 weeks, the participants were using the proposed system and quantitative data of their activity when doing their normal daily activities around city were gathered using the smartphone sensors and Madrid smart city infrastructure.

Data gathered during the first month (30 natural days) after participant’s enrolment were used as individual behavioral pattern baseline. This includes the physical activity, social and leisure and transport behavioral pattern.

Physical activity behavioral pattern was defined by the daily average steps number, daily average distance covered and walking average speed [18, 19]. The social and leisure behavioral pattern was defined by a set of individual points of interest selected by each of the user, according their personal daily routines and preferences, including leisure areas, family members or friends homes, cinemas, theatres, parks or commercial area [20, 21]. In this case, the weekly number of visits and the average visit time duration defined the behavioral pattern. The combination of the physical activity pattern and time expended at home or leisure areas concludes the level of socialization, loneliness, and mood.

Finally, the daily transport usage pattern was defined by the most common urban bus lines, the average distance per trip, the average time spent per trip. The use and how-to-use the public transport conclude the physical and cognitive statuses of the participant. The destination concludes socialization, daily activity patterns and mood [22].

Once, the individual baseline was established, the system collected data from during the participant daily activity. The analysis consisted of detecting gradual or abrupt behavioral changes in these established patterns and conclude links with events that affect the activities. A behavioral pattern change was defined as follows, depending on the data category:

- An activity behavior change was defined as a significant variation of number of steps, distance or walking average speed [5, 23].
- A weekly visits pattern change was defined as a change in the type of visited POIs or in case the POI was not changed, a variation in the duration or frequency of the visit [21].
- A transport daily usage pattern change was defined as a variation in the distance and/or time of usage of transport [22].

In addition, a fourth type of behavior change was included to alert about the lack of data gathering. In this case, the behavioral change was defined as a modification of data gathering pattern for a period of more than 2 weeks (30 natural days). The reason of including 30 days was participants were very active individuals that travel frequently during periods of 10 days or more. During these periods, the data gathering was affected during the week before the trip and the week after trip back. To avoid false positives, we have included this 30-day period as reference period for lack of gathering data.

## Results

A total of 45 participants (20 from daily centers and 25 from SECOT) were finally enrolled in the Madrid pilot deployment. The participant mean age was 79.1 years. Total of women comes from the daily centers cohort, that were slightly but significantly older (mean age 81.2 years) than those from the SECOT group (mean age in SECOT 75.4) and with lower educational level (usually primary studies) and incomes level (810€/month on average on daily center cohort and 1327€/month on average on SECOT cohort). Significantly more participants lived with their partner and every other participant who lives alone was a woman but one. A summary of participants’ characteristics based on sociodemographical data at baseline is presented in Table 2. There was no significant difference between the two groups on other variables.

**Table 2:** Participant baseline demographics

	Total (*n* = 45)		SECOT (*n* = 25)		Daily centers (*n* = 20)	
	*n*	%	*n*	%	*N*	%
Gender						
Man	31	68%	25	100%	6	30%
Woman	14	32%	0	0%	14	70%
Edad						
Average	79.51		75.56		84.45	
< 75	6	13%	6	24%	0	0%
75–80	10	22%	10	25%	0	0%
80–85	18	40%	5	20%	13	65%
85–90	11	25%	4	16%	7	35%
Marital status						
Single	2	5%	0	0%	2	10%
Married	34	75%	25	100%	9	45%
Divorced	0	0%	0	0%	0	0%
Widower	9	20%	0	0%	9	45%
Living condition						
Live alone	10	20%	0	0%	10	50%
Couple without children living at home	24	53%	17	68%	7	35%
Couple with children living at home	9	23%	8	32%	1	5%
Living with his/her children	1	2%	0	0%	1	5%
Living with other relatives (grandchildren, brothers or sisters, etc)	1	2%	0	0%	1	5%
Level of education						
No schooling	1	2%	0	0%	1	5%
Primary studies	10	22%	0	0%	10	50%
Secondary studies	16	35%	14	56%	7	35%
University	13	30%	11	44%	2	10%
Monthly incomes						
< 700€	6	13%	0	0%	6	30%
700–1200€	4	9%	0	0%	4	20%
1200–1900€	16	35%	8	32%	8	40%
1900–2700€	8	18%	8	32%	0	0%
> 2700€	2	4%	2	8%	0	0%
NC	9	20%	7	28%	2	10%

At the end of the pilot (November 2018) the cohort has been followed for a mean of 71 weeks ± 24 weeks. Since baseline, 14 participants (31%) required withdrawal from the project because of diverse causes. Six participants withdrew 3–5 months after enrollment due to uneasiness with the solution and feeling overwhelmed by pilot procedures. Seven withdrawals later on due to health problems and/or familiar (usually their couple) health problems or need of caring a familiar (usually a baby or toddler grandson). Finally, one withdrew because of moving to another city. These participants’ data have not been included in the final analysis results.

There was no significant difference between the two groups in frailty index and general health status. Participants were robust persons according the [9] criteria with 1–3 frailty index at baseline. Participants were also active adults that usually engage with recreational or physical activities of moderate or high intensity 2 or more days (weekly).

None of the participants changed their frailty index during the experiment according the results of the frailty/cognitive assessment tests. Small changes were observed in the results and answers of these tests during the 96 months experiment period, without influence in participants’ independence or robust assessment.

Therefore, important changes in the data gathering patterns were detected during this period in some of the participants. In particular, in 20 of them (60%), we detected changes in the number of steps, walking distance, step speed and walking time against their personal baseline, one or more periods.

In these participants, a reduction, even a total absence, of data gathering, was detected with a trend to recover normal data gathering pattern after 2–6 weeks period. During these periods, participants’ walking distance and step speed were the data more affected in the 65% of the cases, with important reduction in the average walking speed and average walking distance per session. In all of them the POIs visits (type of visited POIs, time expended in each POIs) and the public daily transport usage patterns had also changed.

Since the frailty/MCI assessment tests had not sent indicators of physical/cognitive problem, we had used the data collected from the personal interviews and the medical history to try to explain these behavioral changes.

Data from periods around data loss/data lack had matched with events that impact on participants’ health or their family. About the 65% of these participants had suffered an event that could explain the data behavioral change, mainly an illness (39%) or hospitalization (31%). Other causes were falls (14.3%) and relatives care (14.7%).

In those participants (about the 78%) that had experiment an illness (flu, bronchitis, gastrointestinal disorders mainly, but also lack of energy and asthenia), a significant reduction of leisure-related POIs (32% in average in time weekly visits) together with increasing time at home (58% more of time spent at home) and a higher number of medical-related POIs visits were observed (27% in average). During these periods, participants has also reduced the data gathering volume in 49% to 100%.

The transport behavior patterns were also affected in those participants affected with illness. Some of them (about de 56% of those that suffer an illness) had increased the number of small trips in lines around their neighborhood (26–70% more of trips per day) while others (22%) dropped out the numbers of trips, during the illness and recovery period.

This transport behavior change was also observed in participants (about the 60% of those that have changed their transport behavior) that had taken care of their grandchildren or other family member. Leisure POIs visits were also reduced in this cases, especially daily, with lower incidence on weekends.

## Discussion and implications

With the increased prevalence of age-related diseases, such as frailty, it is of great importance to produce new models that support preventive and personalizable health and care services. The aim of this system is to predict early symptoms of frailty and in this way, to predict adverse health outcomes. These results demonstrate for the first time the feasibility of a system oriented to use the infrastructure of a smart city to detect the absence of early symptoms of frailty. They further show that the urban communities of older adults living in Smart Cities can benefit of their behavioral data gathering while they are at home or in the move within the city in a completely unobtrusive manner. We report here basic continuous activity metrics for an aging population while doing their daily activities in the city (number of steps, distance covered, and walking average speed, type of POI, number of visit per POI, visit duration, number of trips, used bus lines, distance, and time per trip). There are no current standards for reporting these data. Therefore, these continuous monitorings were not designed to be used as a single point for frailty assessment, such as the bimonthly tests. These data have the potential of identifying relevant behaviors of individuals and early detecting changes that can be correlated with risks related to cognitive impartments and frailty, especially illnesses or events that could affect their independence. The 30-day windows balances adequate opportunity to observe routine typical activity around the biweekly self-assessment and changes that usually are not reported to medical services in early stages.

In addition, the continuous monitoring provides the opportunity to capture activity in the typical environment in which each individual lives, without external intervention, and thus is likely to represent a more real measure of real activities and behavior patterns, than self-report tests. This is especially important, for instance, in self-report activity, because it is documented and that seniors perceive they are more active than they are [24, 25].

It is noted that frailty is usually associated to oldest women with lower incomes [26], but in our study, the data demonstrated no differences in active mobility patterns, transport or socialization-related POIs and adverse events between genders, even female participants’ average age is higher than men’s (mean age 81 years in the case of women as compared with 77 years in case of men) and with lower incomes (usually low window’s pensions of 600€ or lower). Further research needs to be explored in this area, to find the mobility factors that influence in the higher prevalence of frailty in this group.

Limitation in the study is that is representative of active and healthy seniors living independent and willing to be monitor with a system like the proposed one. Nevertheless, more than 50% of participants were 80 years or more, suggesting that this group of oldest old, traditionally consider to be lagging in technology, is willing to engage with new technologies if they perceive they can manage their health better [27].

It is noted that participants had little or no functional disability in activities of daily living, whereas the population-based studies, up to 75% of the oldest old will be impaired in one or more activities of the daily living [28]. This is due to the entry requirement for study that stipulate no dependency so that we could follow changes in our metrics over time that would predict the future development of frailty or impairments.

The density of the data and the potential for personal annotation may ultimately provide an opportunity to conduct statistically meaningful “n-of-one” studies.

In summary, the very early detection of events that could impact negatively in physical or/and cognitive status of older citizens could facilitate the developing of ICT-based interventions to mitigate these risks, delaying the ageing functional decline, reverting early stages of frailty and stimulating and providing older adults with incentives to remain active, involved, and engaged. Moreover, this approach could empower citizens to take control of their wellbeing, while policy makers and health professionals could use data to deliver preventive health services and apply specific interventions where they are most needed, enable better management of resources and reducing associated costs.

Ongoing work will focus on refining and resolving the activity recognition approach, combining new data-driven and knowledge-driven techniques that provide to the proposed system with the ability to learn and personalize the expert knowledge target activities. The objective is to allow for personalized activity models that will improve the automatic recognition of behavioral changes and allow larger community-based studies.

In addition, new interaction models should be explored, like the use of smartwatches as main monitoring device, that improve engagement and avoid data loss due to smartphone forgotten at home or discharged battery should be applied, always oriented to avoid depending on smartphone sensors, increasing data usage form smart city infrastructure and their distributed sensor networks and the use wearable devices. This will provide an to opportunity to open many new ways of assessing social engagement to functional decline due to age. The long-term potential of smart aging friendly cities in the utilization to mainstream of assessment of frailty and MCI could achieve true personalized policies of active and healthy ageing and reduce the socio-economic impact of frailty in the social and care systems.
